# Nuclear Phosphoinositide-Specific Phospholipase C β1 Controls Cytoplasmic CCL2 mRNA Levels in HIV-1 gp120-Stimulated Primary Human Macrophages

**DOI:** 10.1371/journal.pone.0059705

**Published:** 2013-03-28

**Authors:** Francesca Spadaro, Serena Cecchetti, Cristina Purificato, Michela Sabbatucci, Franca Podo, Carlo Ramoni, Sandra Gessani, Laura Fantuzzi

**Affiliations:** 1 Department of Hematology, Oncology and Molecular Medicine, Istituto Superiore di Sanità, Rome, Italy; 2 Department of Cell Biology and Neurosciences, Istituto Superiore di Sanità, Rome, Italy; Università degli Studi di Milano, Italy

## Abstract

HIV-1 envelope glycoprotein gp120 induces, independently of infection, the release of CCL2 from macrophages. In turn, this chemokine acts as an autocrine factor enhancing viral replication. In this study, we show for the first time that phosphoinositide-specific phospholipase C (PI-PLC) is required for the production of CCL2 triggered by gp120 in macrophages. Using a combination of confocal laser-scanner microscopy, pharmacologic inhibition, western blotting and fluorescence-activated cell sorter analysis, we demonstrate that gp120 interaction with CCR5 leads to nuclear localization of the PI-PLC β1 isozyme mediated by mitogen-activated protein kinase ERK-1/2. Notably, phosphatidylcholine-specific phospholipase C (PC-PLC), previously reported to be required for NF-kB-mediated CCL2 production induced by gp120 in macrophages, drives both ERK1/2 activation and PI-PLC β1 nuclear localization induced by gp120. PI-PLC β1 activation through CCR5 is also triggered by the natural chemokine ligand CCL4, but independently of ERK1/2. Finally, PI-PLC inhibition neither blocks gp120-mediated NF-kB activation nor overall accumulation of CCL2 mRNA, whereas it decreases CCL2 transcript level in the cytoplasm. These results identify nuclear PI-PLC β1 as a new intermediate in the gp120-triggered PC-PLC-driven signal transduction pathway leading to CCL2 secretion in macrophages. The finding that a concerted gp120-mediated signaling involving both PC- and PI-specific PLCs is required for the expression of CCL2 in macrophages suggests that this signal transduction pathway may also be relevant for the modulation of viral replication in these cells. Thus, this study may contribute to identify novel targets for therapeutic intervention in HIV-1 infection.

## Introduction

CCL2, previously known as monocyte chemoattractant protein-1 (MCP-1), is a member of the CC-chemokine family secreted by a variety of both hematopoietic and non-hematopoietic cells, with monocytes/macrophages representing the major source in the peripheral blood [1], [2]. CCL2 regulates the migration and infiltration of monocytes, CD4^+^ T lymphocytes and NK cells. From a clinical point of view, CCL2 is one of the most studied pro-inflammatory molecules among the chemokine family and represents a potential intervention point for the treatment of various inflammatory, autoimmune and infectious diseases. Interestingly, this chemokine is induced during a variety of human acute and chronic viral infections. Among the viruses inducing CCL2 in humans, HIV-1 has evolved several mechanisms to ensure sustained CCL2 production. In fact, in addition to infection itself, virus-derived proteins such as gp120, Nef, matrix protein p17 and transactivator protein Tat induce a significant increase in the expression and release of this chemokine [3].

In addition to T lymphocytes, monocytes/macrophages represent a primary target and host of HIV-1, and also act as an important reservoir and vehicle of transmission [4], [5]. Tissue macrophages are among the first cells to be infected by the virus. These cells are not subjected to viral-induced death and persist as reservoirs of virus in tissues for long time. Furthermore, macrophages are highly secretory cells which represent an important source for a variety of soluble immune mediators, including cytokines and chemokines, and strongly contribute to the dysregulation of soluble factor production observed at all stages of HIV infection [6], [7].

The complex interactions of human cells with HIV-1 not only include effects restricted to productive infection but also induce responses that extend beyond active viral replication. Among the events following viral exposure that may be unrelated to infection, the greatest effects have been attributed to the envelope glycoprotein gp120. Besides facilitating viral entry, gp120 binding to chemokine receptors in several cell types, including monocytes/macrophages, may also initiate signaling events that may have important implications for pathogenesis by affecting post-entry stages of infection or by modulating cellular functions apart from infection [8], [9]. Cellular signal transduction pathways have been shown not only to be perturbed by HIV infection, but their activation can conversely regulate the replicative capacity of HIV-1. It has been demonstrated that the interaction of virion-associated or soluble gp120 with CCR5 or CXCR4 co-receptors, independently of CD4 engagement, results in receptor-coupled G protein activation and intracellular Ca^2+^ accumulation, leading to Pyk2 activation [10]. The Src kinase Lyn, MAP and PI3 kinases are also activated through gp120 engagement of co-receptors [10]–[14], whereas STAT family members activation is specifically triggered through gp120 interaction with CD4 [15]. Interestingly, the gp120-mediated activation of some of these pathways has been directly correlated with the production of soluble factors, including CCL2 [11], [16]–[18].

Phospholipase-mediated phospholipid hydrolysis is a widespread response elicited by most growth factors, cytokines, hormones, neurotransmitters and other extracellular signals [19]. Phospholipases are a family of enzymes classified according to the phospholipid bond that is cleaved. Phospholipid hydrolysis products include many of the most important second messengers that have been implicated in a wide range of cellular responses. In particular, phosphoinositide-specific phospholipases C (PI-PLCs) play a key role in signal transduction by catalyzing hydrolysis of phosphatidyl-inositol 4,5-bisphosphate to yield two second messengers: inositol-1,4,5-trisphosphate and diacylglycerol, which mediate release of intracellular Ca^2+^ and activation of protein kinase C, respectively [20]. The PI-PLC isozymes are divided into six families: PLC β, γ, δ, ε, ζ and η. Members of each family are distinguished by their modes of activation. These enzymes play an important role in a wide variety of cellular processes and functions, such as proliferation, differentiation, apoptosis, cytoskeleton remodeling, vesicular trafficking, ion channel conductance, endocrine function and neurotransmission [21]. PI-PLCs are soluble proteins mainly localized in the cytosol and, following activation, they translocate to the plasma membrane or to the nucleus, where an independent PI signaling cycle is also present [22]. This nuclear cycle has been shown to be involved in cell proliferation and differentiation, mRNA processing and export [23], [24]. Besides PIs, phosphatidylcholine (PC) is also known to act as substrate for the production of second messengers, mediated by different stimulated phospholipase activities (i.e. PLD, PLC and PLA_2_), involved in receptor-mediated cell signal transduction [25]. Among these enzymes, PC-PLC catalyzes PC hydrolysis into phosphocholine and diacylglycerol. Interestingly, PC-PLC plays a role in basic mechanisms of the immune response, such as cytokine/chemokine production in different experimental systems [18 and ref. herein, 26, 27], cytokine-induced NK cell-mediated cytotoxicity [28], [29] as well as cancer cell proliferation and differentiation [30], [31]. In this context, we previously reported that PC-PLC is required for CCL2 release triggered by HIV-1 gp120 in macrophages [18].

The aim of this study was to investigate the role of PI-PLC in gp120-triggered CCL2 secretion in monocyte-derived macrophages (MDM). We report for the first time that HIV-1 gp120 activates a nuclear PI-PLC β1 signaling pathway involved in the cytoplasmic accumulation of CCL2 mRNA. Triggering of this pathway is mediated by CCR5 coupling to Giα proteins and is driven by PC-PLC through mitogen-activated protein kinase (MAPK) ERK1/2 activation.

## Results

### HIV-1 gp120 Induces Nuclear Localization of the PI-PLC β1 Isozyme in MDM

**Figure 1:**
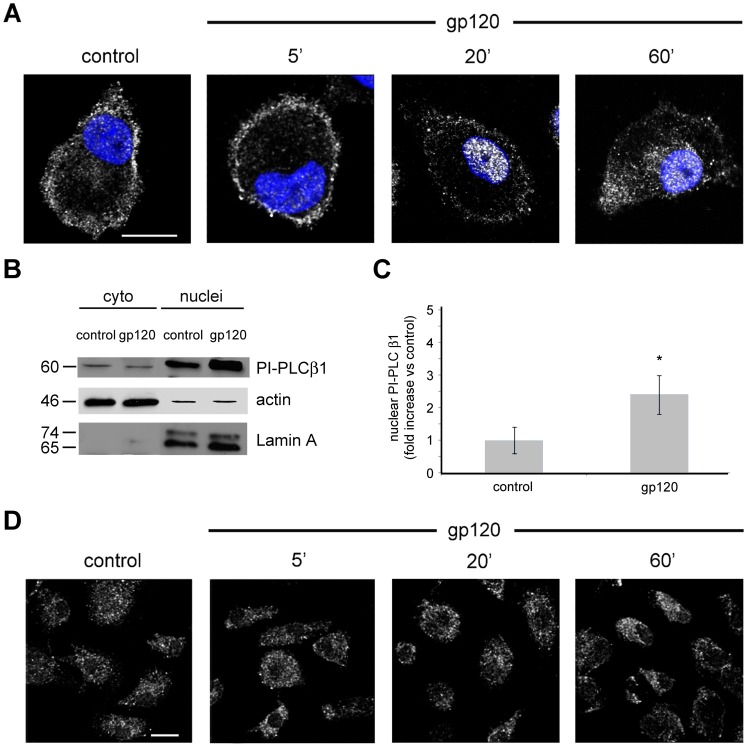
HIV-1 gp120 induces nuclear localization of PI-PLC β1 in MDM. (A) MDM were treated with R5 gp120 (3 µg/ml) for the indicated time periods or left untreated (control). Cells were then fixed, permeabilized, stained with anti-PI-PLC β1 Ab (shown in pseudocolor gray) and examined by CLSM. Nuclei are reported in blue (DAPI). Panels are representative of 4 independent experiments. The bars correspond to 10 µm. (B) MDM were treated with R5 gp120 (3 µg/ml) for 20 minutes and then lysed. PI-PLC β1 expression in cytoplasmic and nuclear extracts was detected by western blotting analysis. Lamin A and β-actin were used as house-keeping control for nuclear and cytoplasmic fractions, respectively. The results from one representative experiment of 4 independently performed are shown. (C) Densitometric analyses of PI-PLC β1 expression performed on immunoblotting of MDM nuclear extracts from the 4 different donors analyzed. *p<0.05. (D) CLSM examinations of cells treated as described in A and then fixed, permeabilized and stained with anti-PI-PLC γ1 Ab (shown in pseudocolor gray). Panels are representative of 4 independent experiments. The bars correspond to 10 µm.

To evaluate whether PI-PLC is involved in the signal transduction pathway triggered by gp120 in MDM, we focused our attention on the β family of PI-PLCs, since the members of this family are activated by heterotrimeric G protein subunits after stimulation of G protein-coupled receptors (GPCRs) [21]. In particular, we investigated the effect of gp120 on cellular localization of the β1 isozyme, the most widely expressed member of the β family of PI-PLCs. By performing confocal laser-scanner microscopy (CLSM) analysis, we found that PI-PLC β1 is highly expressed in control MDM, mainly localized throughout the cytoplasm in proximity to the plasma membrane and poorly in the nucleus (Figure 1A). A short treatment with gp120 (5 minutes) did not substantially modify the distribution of this enzyme, whereas after 20 minutes of gp120 exposure a marked accumulation of the β1 isozyme was observed in the nucleus of MDM (Figure 1A). The β1 nuclear relocalization was found in the majority of the cells, as shown in the following figures. This gp120-induced relocalization was transient, since after 60 minutes of treatment the enzyme appeared to repopulate the cytoplasm around the nucleus (Figure 1A). In agreement with the results of CLSM observations, immunoblotting analysis showed that the amount of the β1 isozyme in purified nuclear extracts increased following 20 minutes of gp120 exposure (Figure 1B). In contrast, a slight decrease in β1 levels was detected in the purified cytoplasmic fractions. To quantify the difference in β1 expression we performed densitometric analyses on immunoblotting of MDM nuclear extracts from 4 different donors, showing that gp120 treatment resulted in a mean fold increase of 2.4±0.6 (SE) in comparison with the non-stimulated control (p<0.05) (Figure 1C). Since the members of the γ family of PI-PLCs, generally regulated by receptor tyrosine kinases, can also be under the control of GPCRs [21], we investigated the effect of gp120 on the γ1 isozyme. As shown in Figure 1D, CLSM analysis revealed that PI-PLC γ1 is highly expressed in the cytoplasm of control MDM but, at variance with what observed for PI-PLC β1, treatment with gp120 did not modify the distribution of this enzyme at any investigated time point.

### PI-PLC Signaling Pathway Mediates HIV-1 gp120-induced CCL2 Production in MDM

**Figure 2:**
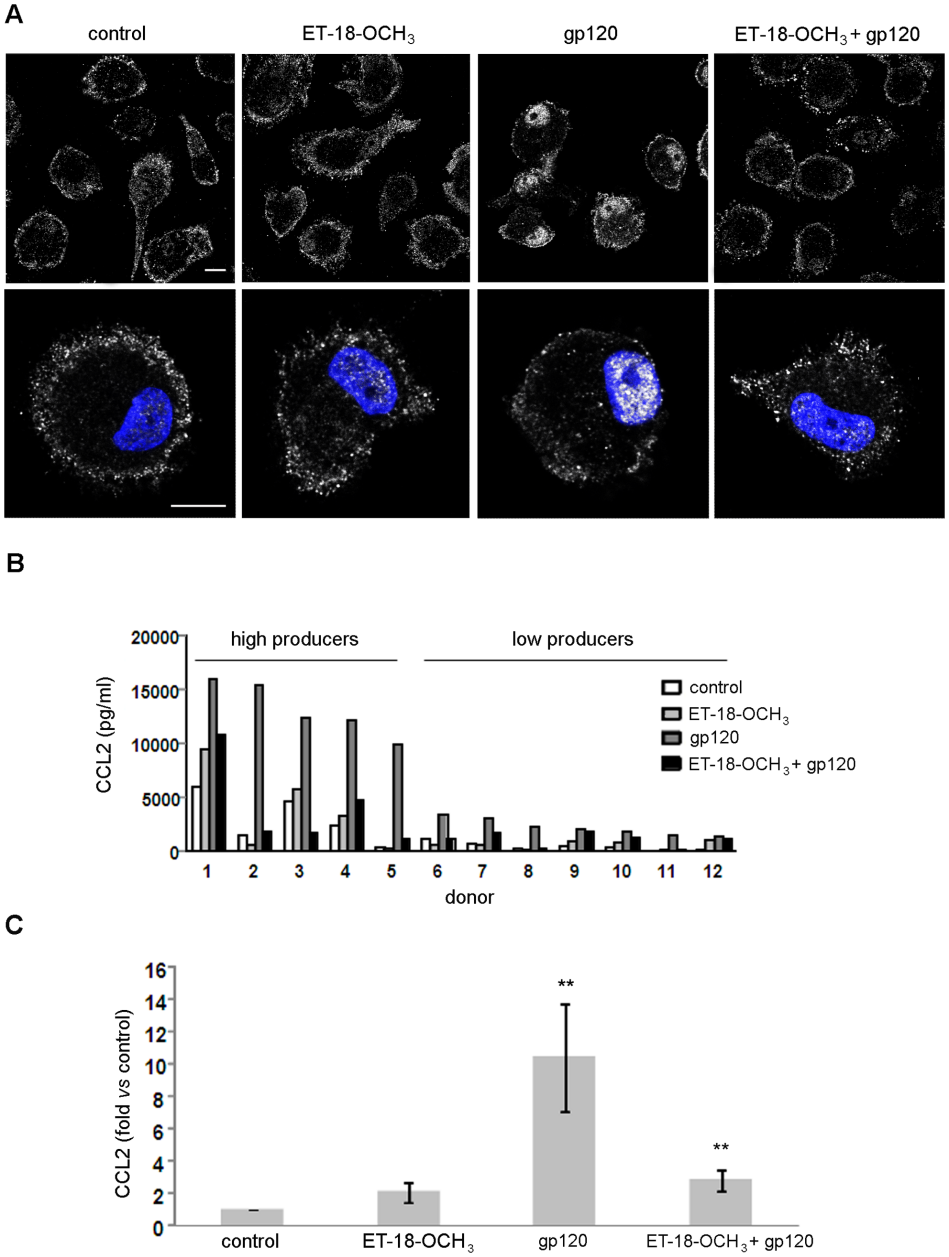
ET-18-OCH_3_ inhibits gp120-mediated PI-PLC β1 nuclear localization and CCL2 secretion in MDM. (A) MDM were incubated with ET-18-OCH_3_ (10 µM) or left untreated. After 1 hour, some cultures were exposed to R5 gp120 (3 µg/ml) for 20 minutes. Cells were then fixed, permeabilized, stained with anti-PI-PLC β1 Ab (showed in pseudocolor gray) and examined by CLSM. Lower panels display a higher magnification of single cells from the corresponding field showed in the upper panels. Nuclei are reported in blue (DAPI). Panels are representative of 5 independent experiments. The bars correspond to 10 µm. (B, C) Monocytes were seeded in 96-well cluster plates. After 7 days, culture medium was removed and replaced with fresh medium. MDM were treated with ET-18-OCH_3_ (10 µM) or left untreated. After 1 hour, some cultures were exposed to R5 gp120 (3 µg/ml) for 24 hours. Supernatants were then harvested and frozen before CCL2 determination. In panel B, the results obtained with all the 12 different donors tested are shown. In panel C, data represent mean values (±SE) of the results obtained with the donors shown in B. **p<0.01 (gp120 *vs* control; ET-18-OCH_3_+gp120 *vs* gp120 alone).

To evaluate the functional consequences of gp120-induced PI-PLC β1 nuclear relocalization, we used the synthetic lysophospholipid analog ET-18-OCH_3_, which acts as a potent and selective PI-PLC inhibitor [32]. Independent experiments showed that this compound did not significantly affect cell viability, as assessed by 3-(4,5-dimethylthiazol-2-yl)-2,5-diphenyltetrazolium bromide (MTT) assay (data not shown). Interestingly, although ET-18-OCH_3_ alone did not significantly modify the distribution of the β1 isozyme in control cells (Figure 2A), it completely inhibited the gp120-induced nuclear accumulation of this enzyme observed after 20 minutes of treatment (Figure 2A). The effect of ET-18-OCH_3_ was not due to a delay in the nuclear localization of the β1 isozyme in response to gp120, since following 60 minutes of stimulation in the presence of the PI-PLC inhibitor, the enzyme still appeared localized in the cytoplasm similarly to control cells, without accumulation in the nucleus (Figure S1).

It has been previously demonstrated that gp120 triggers CCL2 secretion in macrophages [16]–[18]. Furthermore, PI-PLC signaling has been involved in cytokine/chemokine production in different experimental systems [33]–[37]. We therefore investigated whether PI-PLC inhibition could also affect the gp120-induced secretion of CCL2 in MDM. As shown in Figure 2B and C, ET-18-OCH_3_ alone did not significantly modify CCL2 basal secretion. Previous studies reported that some variability in the constitutive expression of CCL2, as well as in the extent of its up-modulation by different stimuli can be observed among donors [17], [18], [38]. As shown in Figure 2B, about 42% of tested donors (5/12) responded to R5 gp120 by secreting high levels of CCL2 (≥10000 pg/ml), whereas about 58% of the donors (7/12) released lower amounts of the chemokine (≤3500 pg/ml). Despite this variability, pre-treatment of MDM with ET-18-OCH_3_ at the concentration of 10 µM inhibited gp120-induced CCL2 secretion. The inhibitory effect of ET-18-OCH_3_ on gp120-induced CCL2 secretion was highly reproducible and consistently observed in all the donors analyzed. In particular, as shown in Figure 2C, a 10.5±3.3 (SE) fold increase in CCL2 secretion was observed in all donors following gp120 stimulation (p<0.01 *vs* control), and this value was reduced to 2.8±0.6 (SE) fold increase upon treatment with ET-18-OCH_3_ (p<0.01 *vs* gp120 alone). Higher doses of ET-18-OCH_3_ (100 µM) similarly affected CCL2 production by gp120, while lower doses (<10 µM) failed to exert any significant effect (data not shown). Similar results were obtained with X4 gp120 (data not shown).

### HIV-1 gp120 Induces PI-PLC β1 Nuclear Relocalization in MDM through CCR5 and Coupling to G_i_α Proteins

**Figure 3:**
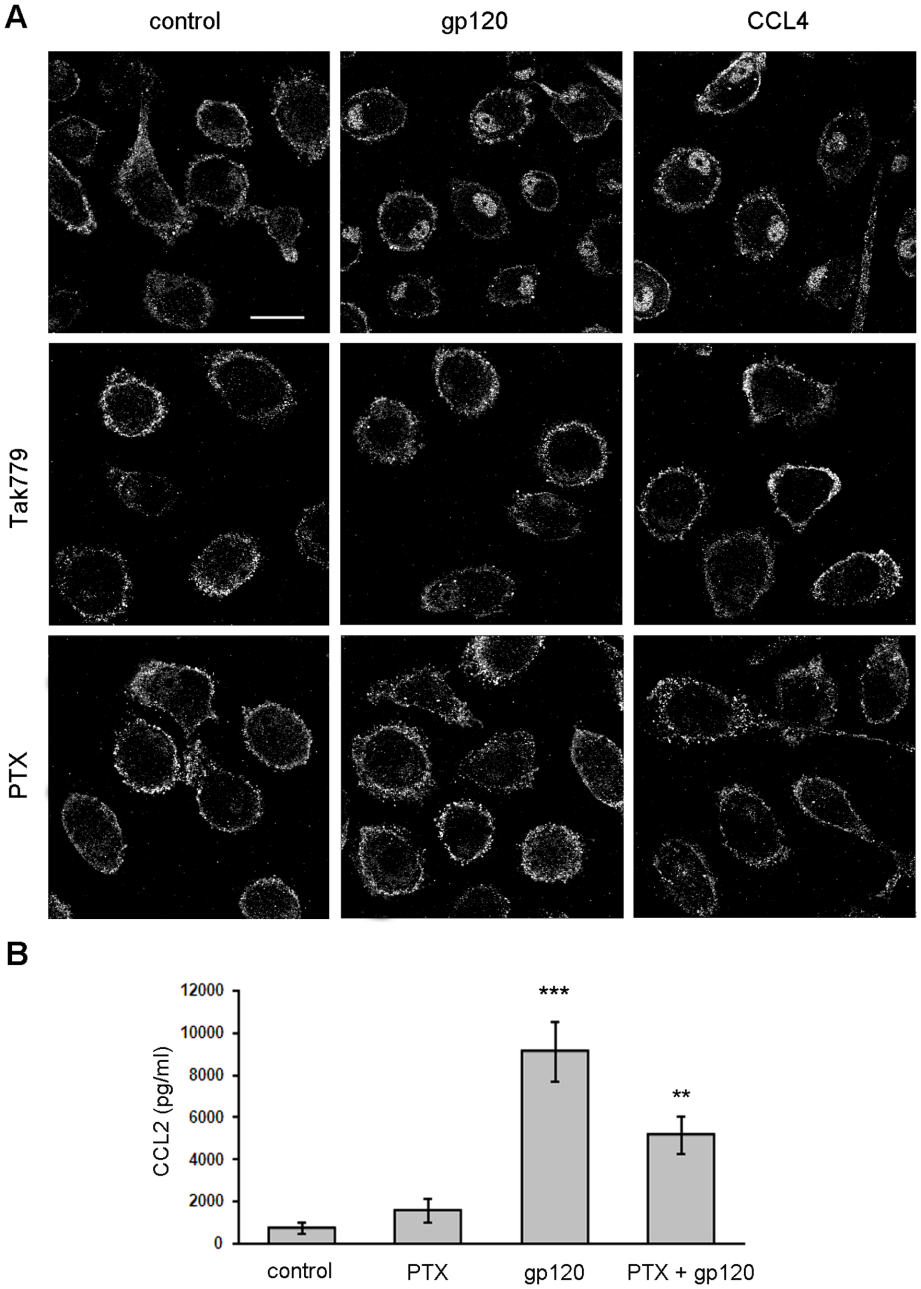
CCR5 and G_i_α inhibitors block R5 gp120-induced nuclear localization of PI-PLC β1 in MDM. (A) MDM were treated with PTX (10 ng/ml) for 2 hours or Tak779 (5 µM) for 1 hour prior to R5 gp120 (3 µg/ml) or CCL4 (200 ng/ml) exposure for 20 minutes. Cells were then fixed, permeabilized and stained with anti-PI-PLC β1 Ab. The cellular distribution of PI-PLC β1 (pseudocolor gray) was monitored by CLSM analysis. Panels are representative of 4 (Tak779) and 3 (PTX) independent experiments. The bars correspond to 20 µm. (B) MDM were treated with PTX (10 ng/ml) for 2 hours prior to R5 gp120 (3 µg/ml) exposure. After 24 hours of culture, supernatants were harvested and frozen before CCL2 determination. Data represent mean values (±SE) of the results obtained with the 9 donors tested. **p<0.01 (PTX+gp120 *vs* gp120 alone); ***p<0.001 (gp120 *vs* control).

We have previously demonstrated that the gp120-mediated secretion of CCL2 in MDM is dependent on its interaction with CCR5 [18]. To define the contribution of this coreceptor in the activation of the PI-PLC β1 isozyme induced by gp120, we used the CCR5-specific pharmacologic antagonist Tak779, previously shown to block HIV-1 coreceptor function, agonist-induced signaling and gp120-mediated CCL2 production [18], [39], [40]. As shown in Figure 3A, MDM pre-treatment with Tak779 completely prevented the nuclear relocalization of the β1 isozyme observed after 20 minutes of R5 gp120 exposure.

It has been shown that macrophage activation by gp120 resulted in CCR5-mediated signals that resembled in some, but not in all aspects those elicited by natural ligands [18], [41]. In this regard, we previously showed that CCL4, the most specific of several chemokines that signal through CCR5, does not activate PC-PLC and CCL2 expression in MDM [18]. We thus investigated whether CCR5 stimulation by CCL4 would elicit PI-PLC β1 activation. As shown in Figure 3A, treatment of MDM with CCL4 for 20 minutes led to nuclear localization of the β1 isozyme similarly to what observed in the presence of R5 gp120, and this effect was blocked by the CCR5 inhibitor Tak779. Furthermore, no substantial differences were observed in the kinetics of β1 cellular relocalization induced by CCL4, compared to gp120 exposure, with a similar maximum nuclear accumulation of the enzyme after 20 minutes of stimulation (Figure S2). Since G_i_α is the main G protein coupled to CCR5 [42], we next investigated the effect of the G_i_α inhibitor PTX, which uncouples G_i_α proteins from G protein-linked 7-transmembrane receptors [43], on stimuli-induced nuclear localization of the β1 isozyme. As shown in Figure 3A, MDM pre-treatment with PTX prevented the nuclear relocalization of PI-PLC β1 observed in response to R5 gp120 and CCL4 as well. In line with these results, PTX significantly reduced the gp120-induced up-modulation of CCL2 secretion (Figure 3B). In particular, the median gp120-triggered secretion in the nine donors analyzed [9148±1414 (SE), p<0.001 *vs* control] was reduced by 44% upon pre-treatment with PTX [5150±891 (SE), p<0.01 *vs* gp120 alone].

### R5 gp120-induced Nuclear Relocalization of PI-PLC β1 Requires MAPK ERK1/2 Activation

**Figure 4:**
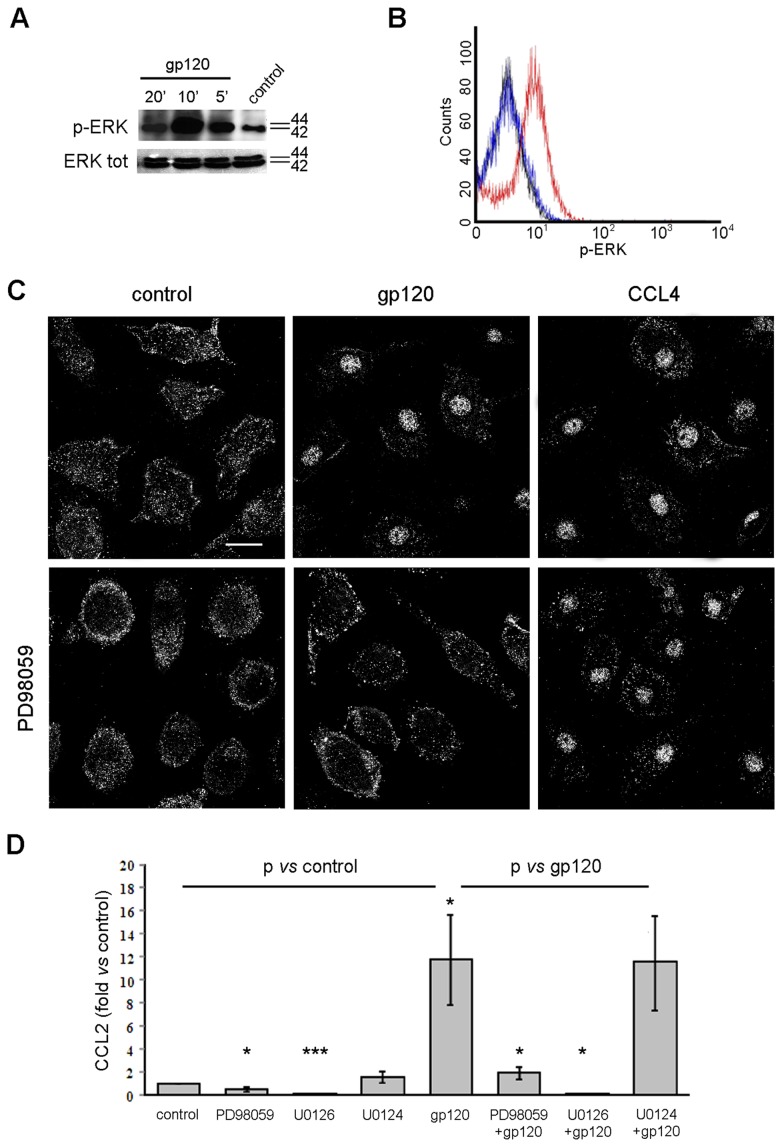
R5 gp120-activated ERK 1/2 mediates nuclear localization of PI-PLC β1 and CCL2 secretion in MDM. (A) MDM were treated with R5 gp120 (3 µg/ml) for the indicated time periods or left untreated (control). Cells were then lysed and ERK 1/2 phosphorylation was detected by western blotting. The results from one representative experiment of 4 independently performed are shown. (B) MDM were treated as described in A and ERK 1/2 phosphorylation was analyzed by flow cytometry after 10 minutes of gp120 exposure. Blue and red lines represent control and gp120-treated cells, respectively. Black line represents background staining of isotype-matched control Ab. The results from one representative experiment of 4 independently performed are shown. (C) MDM were treated with PD98059 (10 µM) for 1 hour prior to R5 gp120 (3 µg/ml) or CCL4 (200 ng/ml) exposure for 20 minutes. Cells were then fixed, permeabilized, stained with anti-PI-PLC β1 Ab (pseudocolor gray) and examined by CLSM. Representative examples of 5 independent experiments are shown. The bars correspond to 20 µm. (D) MDM were treated with PD98059, U0126, U0124 (10 µM) or left untreated (control). After 1 hour, some cultures were exposed to R5 gp120 (3 µg/ml). After 24 hours of culture, supernatants were harvested and frozen before CCL2 determination. Data represent mean values (±SE) of the results obtained with MDM from 10 different donors. *p<0.05; ***p<0.001.

We next wished to dissect the molecular signals involved in gp120-elicited, CCR5-mediated PI-PLC β1 activation in MDM. To this aim, we focused on MAPK ERK1/2, identified as a signaling intermediate in the activation of PI-PLC β1 triggered by several stimuli in different cell models [44]. Furthermore, ERK1/2 has been previously shown to be activated by HIV-1 gp120 in MDM [41], [45]. The involvement of this MAPK in gp120-signaling cascade was first analyzed by western blotting analysis using antibodies able to detect the endogenous phosphorylated and total ERK1/2 proteins, hence determining the kinetics of ERK1/2 activation following exposure of MDM to R5 gp120. As shown in Figure 4A, treatment of MDM with R5 gp120 resulted in a rapid and time-dependent activation of ERK1/2. The peak of ERK1/2 phosphorylation occurred at 10 minutes following gp120 stimulation, afterward decreasing over time. These results were further confirmed by quantitative flow cytometry analysis performed on MDM isolated from different donors. As shown in the representative plot reported in Figure 4B, the activation/phosphorylation status of ERK1/2 in MDM was higher after 10 minutes of gp120 stimulation (red line) compared to non-stimulated MDM (blue line). Among the 6 donors analyzed, the mean percentage of phosphorylated ERK1/2-positive cells following gp120 exposure was 70±13 (SE), compared with 21±6 (SE) of the non-stimulated MDM (p<0.05). We thus investigated whether ERK1/2 was involved in gp120-mediated PI-PLC β1 activation in MDM. As shown in Figure 4C, the ERK1/2 inhibitor PD98059 completely abrogated the nuclear relocalization of PI-PLC β1 observed when MDM were exposed to R5 gp120 for 20 minutes. Similar results were also observed after treatment with a different ERK1/2 inhibitor, namely U0126, but not with its inactive analog U0124 (Figure S3). Interestingly, PD98059 did not affect CCL4-triggered PI-PLC β1 nuclear relocalization (Figure 4C), thus suggesting that different pathways mediate PI-PLC β1 activation in response to gp120 or to chemokine ligands. In keeping with the CLSM results, PD98059, as well as U0126, but not the inactive analog U0124, completely inhibited gp120-induced CCL2 secretion in MDM (Figure 4D). In particular, a 11.7±3.9 (SE) fold increase in CCL2 secretion was observed in MDM of all donors following gp120 stimulation (p<0.05 *vs* control), and this value was reduced to 1.9±0.6 (SE) and 0.2±0.05 (SE) fold increase upon pre-treatment with PD98059 and U0126 (p<0.05 *vs* gp120 alone), respectively. The presence of ERK1/2 inhibitors also significantly reduced the constitutive secretion of CCL2 (Figure 4D). Lower doses of PD98059 and U0126 (1 and 0.1 µM) failed to exert any significant effect on CCL2 production induced by gp120 (data not shown).

### R5 gp120-induced ERK1/2 Activation and Nuclear Relocalization of PI-PLC β1 Requires PC-PLC Activation

**Figure 5:**
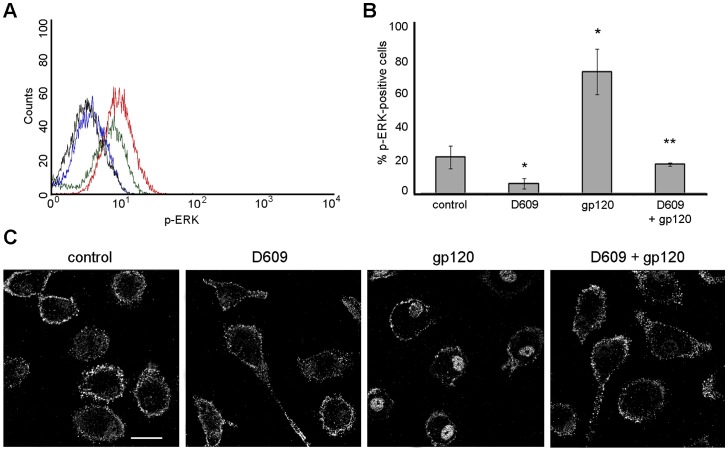
PC-PLC mediates R5 gp120-elicited ERK1/2 phosphorylation and PI-PLC β1 nuclear relocalization in MDM. (A, B) MDM were treated with D609 (50 µg/ml) for 30 minutes prior to R5 gp120 (3 µg/ml) exposure for 10 minutes, and ERK1/2 phosphorylation was evaluated by flow cytometry. In A, a representative histogram from one experiment of 3 independently performed is shown. Green and red lines represent control and gp120-stimulated cells, respectively; black and blue lines represent cells treated with D609 alone or in combination with gp120, respectively. In B, data represent mean values (±SE) of 3 independent experiments. *p<0.05; **p<0.01. (C) MDM were treated with D609 (50 µg/ml) for 30 minutes prior to R5 gp120 (3 µg/ml) exposure for 20 minutes. Cells were then fixed, permeabilized, stained with anti-PI-PLC β1 Ab (pseudocolor gray) and examined by CLSM. A representative experiment of 5 independently performed is shown. The bars correspond to 20 µm.

In our previous study we demonstrated that PC-PLC activation was required for gp120-triggered, CCR5-mediated CCL2 production in MDM [18]. It is worth noting that, in some cell systems ERK1/2 activation is blocked by the selective PC-PLC inhibitor tricyclodecan-9-yl-xanthogenate (D609) [46]–[48]. We thus investigated whether PC-PLC, promptly activated after 5 minutes of gp120 exposure in MDM [18], could mediate gp120-induced phosphorylation of ERK1/2 in these cells. To this aim, MDM were treated with D609 (50 µg/ml) prior to gp120 exposure and ERK1/2 activation/phosphorylation was investigated by flow cytometry. Independent experiments showed that at this dose D609 did not significantly affect MDM cell viability, as assessed by MTT assay (data not shown). As shown in Figure 5A, the presence of D609 (blue and black lines represent D609 alone or in combination with gp120, respectively) markedly reduced both constitutive (green line) and gp120-mediated ERK1/2 phosphorylation (red line). The inhibitory effect of D609 on gp120-induced ERK1/2 activation was highly reproducible and consistently observed in all the donors analyzed. In particular, as shown in Figure 5B, a 3.4±0.7 (SE) fold increase in ERK1/2 phosphorylation was observed in all donors following gp120 stimulation (p<0.05 *vs* control), and this value was reduced to 0.8±0.2 (SE) fold upon treatment with D609 (p<0.01 *vs* gp120 alone). Furthermore, D609 alone significantly reduced the constitutive level of ERK1/2 phosphorylation. These results indicate that PC-PLC is an upstream mediator of ERK1/2 activation in response to gp120.

**Figure 6:**
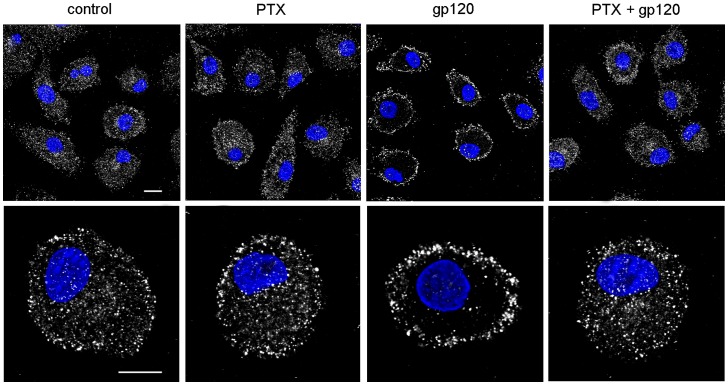
PTX inhibits gp120-induced PC-PLC cellular relocalization in MDM. MDM were treated with PTX (10 ng/mL) or left untreated (control). After 2 hours, some cultures were exposed to R5 gp120 (3 µg/ml) for 5 minutes. Cells were then fixed, permeabilized, stained with rabbit anti-PC-PLC Ab (pseudocolor gray) and examined by CLSM. Nuclei are reported in blue (DAPI). Panels are representative of 3 independent experiments. The bars correspond to 10 µm.

**Figure 7:**
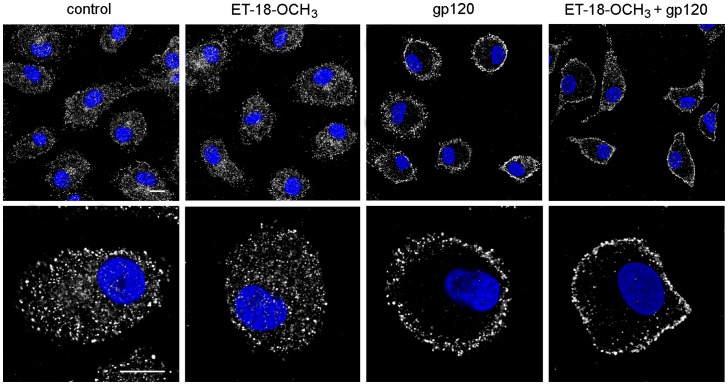
ET-18-OCH_3_ does not affect gp120-induced PC-PLC cellular relocalization in MDM. MDM were treated with ET-18-OCH_3_ (10 µM) or left untreated (control). After 1 hour, some cultures were exposed to R5 gp120 (3 µg/ml) for 5 minutes. Cells were then fixed, permeabilized, stained with rabbit anti-PC-PLC Ab (pseudocolor gray) and examined by CLSM. Nuclei are reported in blue (DAPI). Representative examples of 3 independent experiments are shown. The bars correspond to 10 µm.

As PI-PLC β1 subcellular distribution was strictly dependent on ERK1/2 phosphorylation, we could expect that the gp120-induced nuclear localization of PI-PLC β1 was also mediated by PC-PLC. As shown in Figure 5C, we actually found that D609 completely prevented the nuclear relocalization of PI-PLC β1 observed in response to R5 gp120, thus indicating that the two phospholipases are part of the same signaling cascade triggered through gp120 interaction with CCR5, being PC-PLC an upstream mediator of PI-PLC β1 in this cascade. In keeping with this result, the G_i_α inhibitor PTX, herein shown to block the nuclear relocalization of the β1 isozyme induced by gp120 (Figure 3A), also inhibited the cellular redistribution of PC-PLC, which promptly translocated to the plasma membrane in MDM exposed for 5 minutes to gp120 (Figure 6). Moreover, we found that MDM treatment with ET-18-OCH_3_ prior to gp120 exposure did not affect the PC-PLC cellular relocalization triggered by the viral glycoprotein (Figure 7), further confirming that PC-PLC is an upstream player with respect to PI-PLC β1 in the gp120-triggered, CCR5-mediated signaling cascade leading to CCL2 production in MDM.

### ET-18-OCH_3_ Inhibits the gp120-induced Accumulation of CCL2 Transcripts in the Cytoplasm

**Figure 8:**
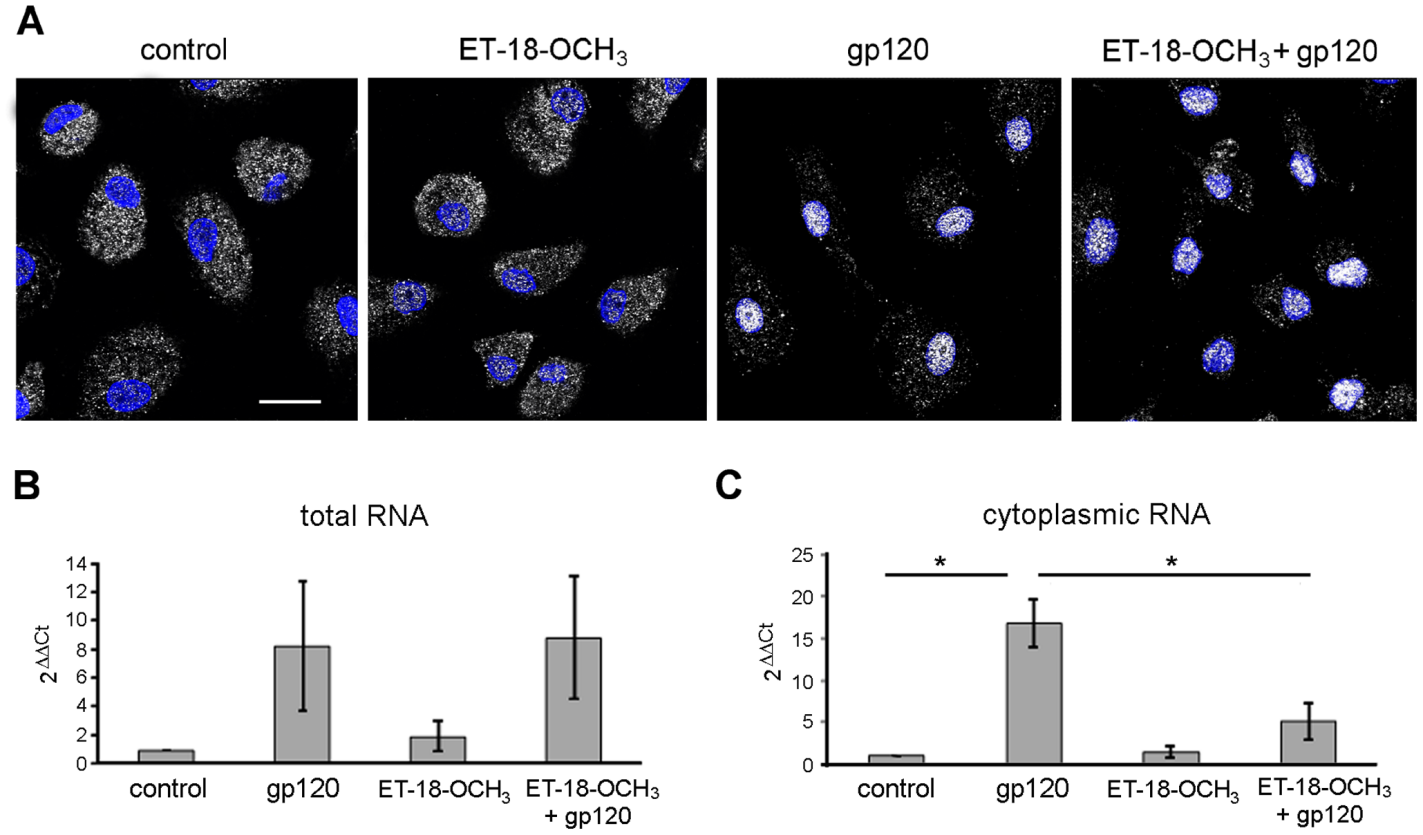
ET-18-OCH_3_ prevents CCL2 transcript accumulation in the cytoplasm of MDM. (A) MDM were treated with ET-18-OCH_3_ (10 µM) or left untreated (control). After 1 hour, some cultures were exposed to R5 gp120 (3 µg/ml) for 1 hour. Cells were then fixed, permeabilized, stained with rabbit anti-p65 Ab (showed in pseudocolor gray) and examined by CLSM. Nuclei are reported in blue (DAPI). Panels are representative of 4 independent experiments. The bars correspond to 20 µm. (B–C) MDM were treated with ET-18-OCH_3_ (10 µM) for 1 hour prior to R5 gp120 (3 µg/ml) exposure for 4 hours. Total (B) or cytoplasmic (C) RNA was then extracted, retrotranscribed and amplified as described in Methods. The 2^∧^(ΔΔCt) values for CCL2 transcripts were calculated as described in Methods. Data represent mean values (±SE) of the results obtained with MDM from 3 different donors. *p<0.05 (gp120 *vs* control; ET-18-OCH_3_+gp120 *vs* gp120 alone).

We previously reported that CCL2 expression in MDM is strongly dependent on NF-kB activation and that PC-PLC is required for R5 gp120-induced NF-kB p65 subunit nuclear translocation and CCL2 transcript accumulation [18]. We thus wondered whether PI-PLC signaling was also involved in NF-kB activation. As shown in Figure 8A, the presence of ET-18-OCH_3_ did not affect NF-kB p65 nuclear localization in MDM either exposed to gp120 for 1 hour or left untreated, clearly demonstrating that PI-PLC is not a component of the gp120-stimulated pathway responsible for NF-kB activation. To provide further evidence on this issue, the effect of ET-18-OCH_3_ on the expression of CCL2 mRNA was assessed by TaqMan real-time RT-PCR. As shown in Figure 8B, R5 gp120 stimulation up-modulated the constitutively expressed CCL2 mRNA, although this effect was not statistically significant among the donors analyzed. Interestingly, treatment with ET-18-OCH_3_ prior to gp120 exposure did not significantly affect CCL2 transcript accumulation, thus adding further evidence that PI-PLC is not a component of the gp120-stimulated, NF-kB-mediated pathway leading to CCL2 transcript accumulation. Since it has been previously shown that nuclear PIs play a critical role in mRNA processing and export [49], [50], and that PI-PLC β1 is the key enzyme involved in the cycle of nuclear PIs [51], we foresaw to determine whether ET-18-OCH_3_ could affect CCL2 mRNA nuclear export in MDM. To this aim, cytoplasmic RNA was extracted from cells treated or not with ET-18-OCH_3_ prior to gp120 exposure. As shown in Figure 8C, when cytoplasmic CCL2 mRNA levels were measured, a significant up-modulation of CCL2 transcript was found in gp120-stimulated MDM. Interestingly, treatment with ET-18-OCH_3_ prior to gp120 exposure resulted in a significant down-modulation of CCL2 transcript accumulation, thus suggesting that PI-PLC β1 was indeed involved in mRNA export from nucleus to cytoplasm.

## Discussion

CCL2 is one of the most important pro-inflammatory products, secreted by immune-activated macrophages, which play a role in the pathogenesis of HIV-1 infection [3], [6]. Previous studies by our and other groups demonstrated a role for this chemokine in the regulation of HIV-1 replication *in vitro* and *in vivo* [52]–[55]. In particular, we found that endogenous CCL2 represents an autocrine factor important for enhancing HIV-1 replication in MDM [55]. The expression and release of this chemokine increase in macrophages infected by HIV-1 or exposed to viral proteins, such as the envelope glycoprotein gp120, Tat and Nef, thus suggesting an immunomodulatory capacity of virus-derived proteins in HIV pathogenesis. Some of the signal transduction pathways involved in gp120-elicited secretion of CCL2 have been, in part, elucidated [16], [18]. In this regard, we previously reported that PC-PLC activation is required for CCR5-dependent, NF-kB-driven CCL2 secretion elicited in response to HIV-1 gp120 in primary human macrophages [18]. In the present study we further dissect the gp120-triggered signaling pathway leading to CCL2 production in these cells. Our results clearly demonstrate that nuclear PI-PLC β1 translocation is induced by gp120 and is required for CCL2 expression and release by controlling the nucleus to cytoplasm transport of CCL2 mRNA. Moreover, we found that this process is mediated by gp120 interaction with CCR5, and is coupled, at least in part, to G_i_α proteins. Furthermore, this pathway requires PC-PLC-driven activation of the MAPK ERK1/2, which represent an intermediate component of this signal transduction cascade, in turn, responsible for the activation of downstream signaling mediators.

An important aspect of the immunopathogenesis of AIDS is represented by the observation that HIV target cells can also be exposed to viral gene products, expressed at the surface of infected cells or released in the microenvironment as a consequence of the death of infected cells. In particular, it is worth mentioning that HIV-1 gp120 is released in tissues and blood of HIV-infected subjects, and it is thought to have a role in the progressive immune derangement observed in these patients [8]. The availability of gp120 in AIDS patients has been documented in a number of reports, either as free protein or complexed by anti-gp120 Abs, suggesting that there could be ample opportunity for virus-associated or shed gp120 to interact with surface components of immune cells. In particular, Oh and coworkers found that the amount of gp120 released in the serum of HIV-infected patients ranged from 12 to 92 ng/ml [56]. In this regard, it must be taken into consideration that any estimation of how much gp120 is naturally present in the HIV-1 positive sera is highly problematic. In fact, the uncertainty about the efficiency of gp120 capture, the extent of cross-reactivity of the detecting Abs with any gp120 present in plasma, and the interference by plasma anti-gp120 Abs, all preclude any accurate estimate of plasma gp120 concentrations. Our previous data showed that 300 ng/ml of gp120 significantly enhanced the production of CCL2 in MDM [17]. Furthermore, we reported that exposure of MDM to R5 gp120 resulted in a concentration-dependent increase of PC-PLC enzymatic activity [18]. This effect was detected with gp120 concentrations ranging from 1 to 3 µg/ml, with a maximal activation at the concentration of 3 µg/ml. Since PC-PLC activation is upstream to PI-PLC β1 nuclear translocation, as we demonstrate in the present manuscript, we used the concentration of gp120 which gives the maximal activation of PC-PLC for the experiments reported here. We envisage that gp120 concentrations suitable for the induction of CCL2, and hence for the triggering of the signaling cascade involved, can be locally achieved in vivo, thus contributing to the up-regulation of CCL2 production observed.

In recent years, a distinct PI cycle has been identified in the nuclear compartment. This nuclear PI pool, independently regulated from the plasma membrane counterpart, plays a critical role in nuclear functions and regulates several cellular processes such as chromatin remodeling, DNA replication and repair, transcription regulation, and RNA dynamics [22], [57]. PI-PLC β1 is the most extensively studied PLC isoform in the nuclear compartment and a key player in the regulation of nuclear lipid signaling [58], [59]. PLC-β differs from the other isozymes γ and δ since it contains a long COOH-terminal sequence that contributes to its translocation and association with the nucleus [60], [61]. Nuclear PI-PLC β1 has been shown to be involved in cell cycle progression and differentiation in response to growth factor stimulation, and recent findings also suggested that it is involved in cancer pathogenesis and progression [62]. To the best of our knowledge, this study provides the first evidence that HIV-1 gp120 triggers a PI-PLC β1-driven nuclear signaling in macrophages. Our results, in fact, clearly demonstrate that PI-PLC β1, generally widely distributed throughout the cytoplasm, localizes to the nucleus following gp120 exposure. In this regard, previous studies performed in different cell systems indicated that PI-PLC β1 sub-cellular localization functions as a major regulatory mechanism of its enzymatic activity [62]–[65]. In particular, Aisiku and coworkers showed that nuclear localization of the β1 isozyme correlates with a high PI-PLC activity in the nucleus [63]. Our findings also suggest that the effect of gp120 on PI-PLCs may be specific for the β1 isoform. In fact, PI-PLC γ1, highly expressed and widely distributed throughout the cytoplasm of macrophages, neither translocates to the plasma membrane nor to the nucleus following gp120 treatment. However, these findings cannot rule out the possibility that other PI-PLC isozymes may be involved in the gp120-triggered signaling cascade. The effects of HIV-1 and its related proteins on PI-PLCs have been poorly investigated. In this respect, Zauli and colleagues [66] showed that the HIV-1 regulatory protein Tat stimulates a PI-PLC nuclear pathway in Jurkat T cell line. However, neither the molecular mechanism responsible for this activation nor its biological significance was elucidated. Interestingly, it has been recently reported that HIV envelope interaction with CCR5 activates different PKC isoforms, which are required for virus entry and replication [67], [68]. It is conceivable that activation of PKC is directly downstream of PI-PLC β1, thus the demonstration that gp120 triggers activation of the β1 isozyme could provide a plausible explanation for the virus-mediated PKC activation. Overall, in this scenario the present study provides new evidence for the HIV-1-mediated molecular mechanism leading to nuclear PI-PLC activation and hence to the signaling pathway triggered by the virus.

To dissect the biological consequences of gp120-mediated PI-PLC β1 activation, we took advantage of ET-18-OCH_3_, the most potent and selective PI-PLC inhibitor so far available [32]. Our data clearly demonstrate that pre-treatment with this drug prevents the PI-PLC β1 nuclear localization triggered by gp120, and also completely abolishes the capacity of the envelope protein to stimulate CCL2 release in macrophages. Interestingly, by measuring CCL2 mRNA levels either in total or cytoplasmic RNA, we demonstrate that the inhibition of PI-PLC does not affect the amount of CCL2 mRNA in the former, whereas it significantly reduces CCL2 transcripts in the latter. Due to the low amount of primary cells available, our efforts to measure CCL2 transcripts in the nucleus have not been successful (data not shown). However, the marked reduction of CCL2 mRNA level in the cytoplasm in the presence of ET-18-OCH_3_ strongly suggests an involvement of nuclear PI-PLC β1 in the export of CCL2 transcripts from the nucleus to the cytoplasm. Furthermore, the herein reported results are in agreement with previous studies suggesting a role for nuclear PI signaling in the regulation of mRNA nucleo-cytoplasmic transport [49], [50]. Concerning the role of PI-PLC in HIV-1 infection, previous studies reported an impairment of both HIV-1 entry [67] and Gag release [69], [70] following inhibition of PI-PLC activity by U73122. Low doses of this compound, shown to be specific for PI-PLC, have been used in these studies, and we found that the same doses of U73122 inhibited gp120-stimulated CCL2 secretion in macrophages (data not shown). Furthermore, alkylphospholipid compounds, including ET-18-OCH_3_, have been recently reported to induce the death of HIV-1-infected primary macrophages and to reduce viral production in these cells [71]. Further investigation of the effect of PI-PLC inhibitors on HIV-1 replication in macrophages is needed to better define the roles played by these enzymes in HIV-1 infection of these cells.

One of the major findings of this study is that PC-PLC plays a key role as an upstream regulator of PI-PLC β1 activation triggered by gp120. Our results, in fact, clearly demonstrate that PI-PLC β1 nuclear localization triggered by gp120 is prevented by pre-treatment with D609. This molecule is thus far the only available compound acting as a competitive PC-PLC inhibitor [72]. Besides acting on PC-PLC, D609 has also been reported to affect PC-PLD activity and to inhibit sphingomyelin synthase in some cell types [73]. However, strong evidence for the specificity of D609 inhibitory effect on PC-PLC has been provided in our previous studies [18], [28], [31]. To the best of our knowledge, this is the first evidence of a direct involvement of PC-PLC in PI-PLC activation, suggesting the existence of a cross-talk between PI and PC cycles. It was previously shown that PLD, the other phospholipase acting on PC headgroup generating phosphatidic acid, also stimulates PI-PLC β1 activity [74]. Thus, our data provide a new link between PC- and PI-specific phospholipase activities and represent one of the few examples of signaling cascades directly involving the action of different phospholipases. In this regard, Andrei and colleagues previously reported that in human primary monocytes LPS-activated PC-PLC enhances intracellular Ca^2+^, which in turn activates cPLA_2_ and lysosomal exocytosis, finally leading to IL-1β release [75]. Furthermore, we identified the MAPK ERK1/2 as a signaling component linking PC-PLC to PI-PLC β1 nuclear translocation. In this regard, Vitale and coworkers previously reported that inhibition of ERK1/2 by PD98059 prevents IL-2-driven nuclear PI-PLC β1 activation [64]. It is noteworthy that PC-PLC inhibition not only prevents the gp120-induced ERK1/2 phosphorylation, but also drastically decreases the constitutive phosphorylation of this MAPK. This suggests that PC-PLC might work at the convergence point of the signaling pathways that regulate both basal and gp120-induced CCL2 expression. Furthermore, we also found that ERK1/2 is required for PC-PLC-driven NF-kB activation, as demonstrated by the inhibitory effect of PD98059 on gp120-mediated NF-kB p65 nuclear translocation (Figure S4). The involvement of this PC-PLC/ERK1/2-driven signaling pathway in NF-kB activation is further strengthened by the finding that CCR5 and G_i_α protein inhibition by Tak779 and PTX, respectively, herein shown to prevent PC-PLC as well as PI-PLC β1 cellular relocalization (Figures 6 and 3), completely blocked gp120-mediated NF-kB p65 nuclear translocation (Figure S5). Overall, our data demonstrate that PTX prevents the activation of this gp120-elicited, CCR5-dependent, PC-PLC-driven pathway. However, PTX treatment only partially inhibited gp120-mediated CCL2 secretion, thus suggesting that induction of this chemokine by gp120 is only in part coupled to G_i_α proteins. In this regard, both PTX-sensitive and PTX-insensitive signaling through CCR5 has been reported for gp120. In particular, Del Corno and coworkers showed that Pyk2 and p38 MAPK activation triggered by gp120 in macrophages is insensitive to PTX, and that p38 activation is involved in gp120-induced CCL2 secretion [16]. Thus, our results, together with those reported in this previous study, suggest that gp120-elicited, CCR5-dependent signaling in macrophages is coupled to G_i_α as well as to G proteins other than G_i_α, and that these different pathways contribute to CCL2 production induced by gp120.

**Figure 9:**
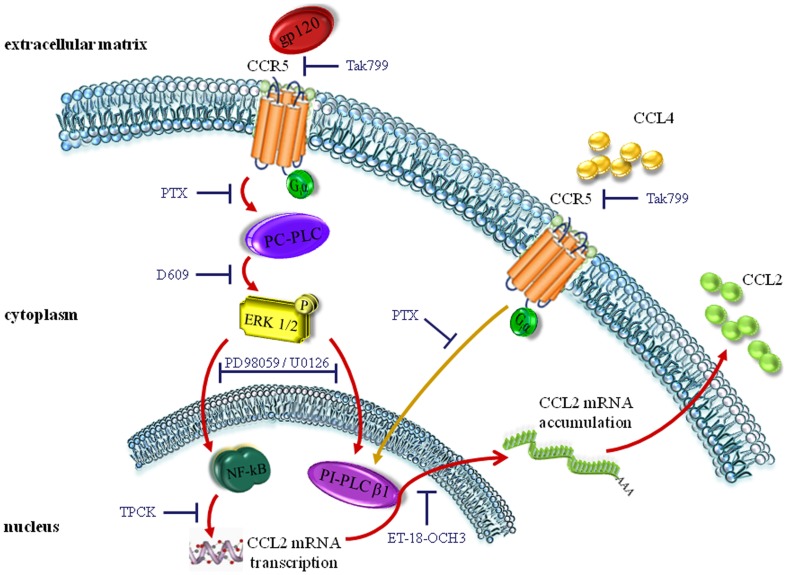
Model for PLC signaling pathway mediating R5 gp120-induced CCL2 production in primary human macrophages. Binding of gp120 to CCR5 triggers PC-PLC-driven activation of ERK1/2 MAPK, required for NF-kB and nuclear PI-PLC β1 activation, ultimately leading to CCL2 transcription and nuclear export of CCL2 mRNA. The natural CCR5 ligand CCL4 activates nuclear PI-PLC β1 in an ERK1/2 independent way, but does not trigger PC-PLC and NF-kB and does not induce CCL2 expression.

The new data provided by the present study, together with those reported in our previous paper, are summarized in the model depicted in Figure 9. In this model, binding of soluble or virion-associated gp120 to macrophage CCR5 triggers G_i_α-mediated activation of PC-PLC. This enzyme, in turn, mediates phosphorylation of the MAPK ERK1/2, which represents a central intermediate driving the activation of the downstream molecules involved in this pathway, such as NF-kB and nuclear PI-PLC β1. The activation of these two signaling molecules is then responsible for CCL2 mRNA transcription and nuclear export, respectively. This PC-PLC-driven signaling pathway is not activated by the natural CCR5 chemokine ligand CCL4. In fact, this chemokine does not activate PC-PLC. Conversely, CCL4 triggers PI-PLC β1 nuclear localization through CCR5 and coupling to G_i_α proteins, but in an ERK1/2 independent way. Despite this effect on the β1 isozyme, CCL4 does not trigger CCL2 secretion, probably because it does not induce NF-kB p65 nuclear translocation (Figure S6). It is well known that HIV-1 gp120 elicits various intracellular signaling events both in primary cells and cell lines that are similar but not identical to that caused by chemokines [10]. In this regard, we demonstrated that gp120, but not CCL4, activated PC-PLC in macrophages [18]. In this previous study, we also reported that co-stimulation of MDM with CCL4 and a monoclonal antibody to CD4, mimicking gp120 binding, does not result in PC-PLC activation. Thus, other mechanisms and/or differential interaction with other cell components may explain the differences in the signalling pathways triggered by these molecules. Overall, our results suggest that different pathways activated through HIV-1 or natural ligand interaction with the same receptor converge on PI-PLC β1 nuclear translocation, but then diverge leading or not to CCL2 secretion, respectively. It is also plausible to speculate that CCL4 might hinder CCL2 production in response to HIV envelope by inducing the internalization of CCR5, thus preventing the triggering of the signaling cascade responsible for CCL2 expression. The identification of a concerted action of different PLCs as novel signaling molecules mediating some of the gp120 biological effects unravels a new mechanism by which HIV-1 may deregulate macrophage functions and contribute to AIDS pathogenesis. This signal transduction pathway is required for expression of CCL2, a chemokine playing an important role in AIDS pathogenesis by recruiting CCR2 positive cells as new targets for infection, contributing to immune activation and enhancing viral replication both in macrophages and T lymphocytes. Therefore, this PLC-mediated signal transduction pathway may contribute to AIDS pathogenesis by controlling the recruitment of new target cells for infection, dysregulating macrophage functions and modulating macrophage and lymphocyte infection. Since inhibition of PI-PLC activity has been previously shown to inhibit viral replication, this signaling cascade might provide potential targets for therapeutic intervention in HIV-1 infection.

## Materials and Methods

### Ethics Statements

Healthy donor buffy coats were obtained from Centro Trasfusionale University of Rome “Sapienza”. Buffy coats were not obtained specifically for this study. Informed consent has not been asked because data were analyzed anonymously. Data from healthy donors have been treated by Centro Trasfusionale according to the Italian law on personal data management “Codice in materia di protezione dei dati personali” (Testo unico D.L. June 30, 2003 n. 196).

### Cell Separation and Culture

Monocytes were isolated from peripheral blood mononuclear cells obtained from healthy donor buffy coats by immunomagnetic selection using CD14 micro beads (MACS monocyte isolation kit from Miltenyi Biotec), according to the manufacturer’s instructions. This procedure yields a 95–98% pure population of monocytes, as assessed by fluorescence-activated cell sorter analysis of lineage-specific surface markers (CD1a, CD14, CD3, CD19, CD56). Monocyte-derived macrophages (MDM) were obtained after 7 days of *in vitro* culture in endotoxin-free Iscove’s medium (Life Technologies) containing 10% FBS as previously described [76].

### Reagents

Recombinant HIV-1 R5 gp120 (primary isolate CN54) was purchased from Dr. I. Jones. As indicated in the data sheet, this protein binds to CD4. Tak779 was obtained from the National Institutes of Health AIDS Research and Reference Reagent Program. Tricyclodecan-9-yl-xanthogenate (D609), 1-O-Octadecyl-2-O-methyl-*rac-*glycero-3-phosphorylcholine (ET-18-OCH_3_), 2′-Amino-3′-methoxyflavone (PD98059), 1,4-Diamino-2,3-dicyano-1,4-*bis* (2-aminophenylthio)butadiene (U126), 1,4-Diamino-2,3-dicyano-1,4-*bis*(methylthio)butadiene (U124) and pertussis toxin (PTX) were purchased from Sigma-Aldrich. All the inhibitors utilized in the experiments did not exhibit any toxicity at the used concentrations, as assessed by MTT assay (data not shown). Recombinant CCL4 was purchased from R&D Systems. The rabbit polyclonal anti-PC-PLC Ab, raised against bacterial (*Bacillus cereus*) PC-PLC and cross-reacting with mammalian PC-PLC, was obtained, purified and characterized as previously described [77], [78]. Mouse anti-PI-PLCβ1 Ab was purchased from Upstate. The rabbit polyclonal anti-NF-kB p65 Ab was purchased from Santa Cruz Biotechnology. The Alexa Fluor 594 conjugated goat anti-rabbit and goat anti-mouse IgG were purchased from Molecular Probe. LPS contamination of reagents was excluded by checking their endotoxin activity by the *Limulus Amoebocyte Assay* (Charles River Endosafe; detection limit 0.125 endotoxin U/ml). The endotoxin content determined in gp120 preparations was below the detection limit of the assay.

### Measurement of CCL2

The levels of CCL2 (detection limit 5 pg/ml) present in culture supernatants were measured by enzyme-linked immunosorbent assay (ELISA) kits purchased from R&D Systems.

### Confocal Laser-scanner Microscopy (CLSM) Analysis

For CLSM analysis, freshly isolated monocytes were seeded in 24-well cluster plates on cover glasses (diameter, 12 mm; 2×10^5^ cells per well in 1 ml). After 6 days, culture medium was removed and replaced with serum-free medium to maintain the cells in a quiescent state. After 24 hours, cells were exposed to the different stimuli for the indicated time periods. The cover glasses were then extensively washed with phosphate-buffered saline (PBS), fixed, permeabilized and stained with mouse anti-PI-PLC β1, rabbit anti-PC-PLC or rabbit anti-p65 Abs, followed by fluorochrome-conjugated secondary Abs, as previously described [18]. Cover glasses were finally mounted on the microscope slides with Vectashield antifade medium containing DAPI (Vector Laboratories Inc.). CLSM observations were performed on a Leica TCS SP2 AOBS apparatus (Leica Microsystemsy), using 63X/1.4 NA oil objective and excitation spectral laser lines at 405 and 594 nm and using the confocal software (Leica) and Photoshop CS2 (Adobe Systems). Cells stained only with the fluorochrome-conjugated secondary antibodies were used to set up acquisition parameters. Different fields were analyzed for each labeling condition, and representative results are shown.

### Western Blotting Analysis

Freshly isolated monocytes were seeded in 6-well cluster plates (6x10^6^ cells per well in 3 ml). After 6 days, culture medium was removed and replaced with serum-free medium to maintain the cells in a quiescent state. After 24 hours, cells were subjected to the different treatment for the indicated time periods. For PI-PLC β1 protein expression studies, cytoplasmic and nuclear extracts were isolated by using a nuclear extract kit (Active Motif), following the manufacturer’s instructions. Lysate (15 µg) was resolved by SDS-PAGE and blotted with mouse anti-PI-PLC β1, mouse anti-β-actin (Sigma-Aldrich) and mouse anti-lamin A (Millipore) antibodies to ensure the quality of protein separation and loading. For ERK1/2 activation studies, cells were lysed in radio immunoprecipitation assay buffer (150 mM NaCl, 50 mM Tris-Cl, pH 7.5, 1% Nonidet P-40, 0.5% sodium deoxicholate, 0.1% SDS) containing the complete protease inhibitor cocktail (Roche Molecular Biochemicals) and a mix of 1 mM Na_3_VO_4_ and 20 mM NaF. Lysate (50 µg) was resolved by SDS-PAGE and blotted with mouse anti-phospho ERK1/2 (Cell Signaling) and rabbit anti-total ERK1/2 (Sigma Aldrich) antibodies. Protein concentrations were determined by protein assay (Bio-Rad Laboratories).

### Flow Cytometry Analysis of ERK1/2 Phosphorylation

Freshly isolated monocytes were seeded in 24-well cluster plates (2.5×10^6^ cells per well in 2.5 ml). After 6 days, culture medium was removed and replaced with serum-free medium to maintain the cells in a quiescent state. After 24 hours, cells were subjected to the different treatment for the indicated periods of time. Cells were then detached by using cold PBS on ice for 30 minutes, recovered and p-ERK expression was evaluated by flow cytometry. Briefly, cells were fixed by adding formaldehyde to cell suspensions at 37°C, followed by permeabilization in 90% ice-cold methanol. After 30 minutes on ice, cell suspensions were centrifuged and washed once with PBS containing 4% bovine serum albumin (Sigma Aldrich) and then labeled with the primary monoclonal Ab specific for p-ERK. An irrelevant mouse Ab of the appropriate subclass was used as a negative control to determine background fluorescence. Cells were then washed and incubated with a goat Ab fragment anti-mouse IgG conjugated with phycoerythrin (Molecular Probes) and samples were analyzed immediately by a FACSCalibur (BD Biosciences).

### Analysis of CCL2 mRNA by TaqMan Real-time RT-PCR

Freshly isolated monocytes were seeded in 24-well cluster plates (2.5×10^6^ cells per well in 2.5 ml). After 6 days, culture medium was removed and replaced with serum-free medium to maintain the cells in a quiescent state. After 24 hours, cells were subjected to the different treatments for 4 hours. Cells were then lysed and total or cytoplasmic RNA was isolated with the RNeasy Mini kit (Qiagen) or the cytoplasmic/nuclear RNA purification kit (Norgen), respectively, following the manufacturer’s instructions. RNA was retrotranscribed and cDNA subjected to real-time PCR as previously reported [18]. Relative quantification was performed by using the comparative Ct method as previously described [18].

### Statistical Analysis

Statistical analysis was performed using the paired Student’s t test. Values of p<0.05 were considered significant.

## Supporting Information

Figure S1 **Time-dependent effect of ET-18-OCH_3_ on gp120-induced PI-PLC β1 nuclear localization in MDM.** MDM were treated with ET-18-OCH_3_ (10 µM) or left untreated (control). After 1 hour, some cultures were exposed to R5 gp120 (3 µg/ml) for the indicated time periods. Cells were then fixed, permeabilized, stained with anti-PI-PLC β1 Ab (pseudocolor gray) and examined by CLSM. Nuclei are reported in blue (DAPI). Representative examples of 3 independent experiments are shown. The bars correspond to 10 µm.
(TIF)

Figure S2 **Time-dependent effect of CCL4 on PI-PLC β1 nuclear localization in MDM.** MDM were treated with CCL4 (200 ng/ml) for the indicated time periods or left untreated (control). Cells were then fixed, permeabilized, stained with anti-PI-PLC β1 Ab (pseudocolor gray) and examined by CLSM. Nuclei are reported in blue (DAPI). A representative experiment of 4 independently performed is shown. The bars correspond to 10 µm.
(TIF)

Figure S3 **The ERK1/2 inhibitor U0126 abrogates gp120-induced PI-PLC β1 nuclear localization in MDM.** MDM were treated with U0126 or its inactive analog U0124 (10 µM) or left untreated (control). After 1 hour, some cultures were exposed to R5 gp120 (3 µg/ml) for 20 minutes. Cells were then fixed, permeabilized, stained with anti-PI-PLC β1 Ab (showed in pseudocolor gray) and examined by CLSM. Panels are representative of 3 independent experiments. The bars correspond to 20 µm.
(TIF)

Figure S4 **PD98059 inhibits gp120-induced NF-kB p65 subunit nuclear translocation in MDM.** MDM were treated with PD98059 (10 µM) or left untreated (control). After 1 hour, some cultures were exposed to R5 gp120 (3 µg/ml) for 1 hour. Cells were then fixed, permeabilized and stained with rabbit anti-p65 Ab (showed in pseudocolor gray) and examined by CLSM analysis. Nuclei are reported in blue (DAPI). The results are representative of 4 independent experiments. The bars correspond to 20 µm.
(TIF)

Figure S5 **Tak779 and PTX prevent gp120-induced NF-kB p65 subunit nuclear translocation in MDM.** MDM were treated with PTX (10 ng/ml) for 2 hours or Tak779 (5 µM) for 1 hour prior to R5 gp120 (3 µg/ml) exposure for 1 hour or left untreated (control). Cells were then fixed, permeabilized and stained with rabbit anti-p65 Ab (showed in pseudocolor gray) and examined by CLSM analysis. Nuclei are reported in blue (DAPI). The results are representative of 2 independent experiments. The bars correspond to 20 µm.
(TIF)

Figure S6 **CCL4 does not induce NF-kB p65 subunit nuclear translocation in MDM.** MDM were treated with R5 gp120 (3 µg/ml) or CCL4 (200 ng/ml) or left untreated (control). After 1 hour, cells were fixed, permeabilized and stained with rabbit anti-p65 Ab (showed in pseudocolor gray) and examined by CLSM analysis. Nuclei are reported in blue (DAPI). Panels are representative of 2 independent experiments. The bars correspond to 20 µm.
(TIF)
