# Supplementary figures and images for: Nuclear Phosphoinositide-Specific Phospholipase C β1 Controls Cytoplasmic CCL2 mRNA Levels in HIV-1 gp120-Stimulated Primary Human Macrophages
Source: PLoS One. 2013 Mar 28;8(3):e59705. doi: 10.1371/journal.pone.0059705 (PMC3610878; doi:10.1371/journal.pone.0059705)

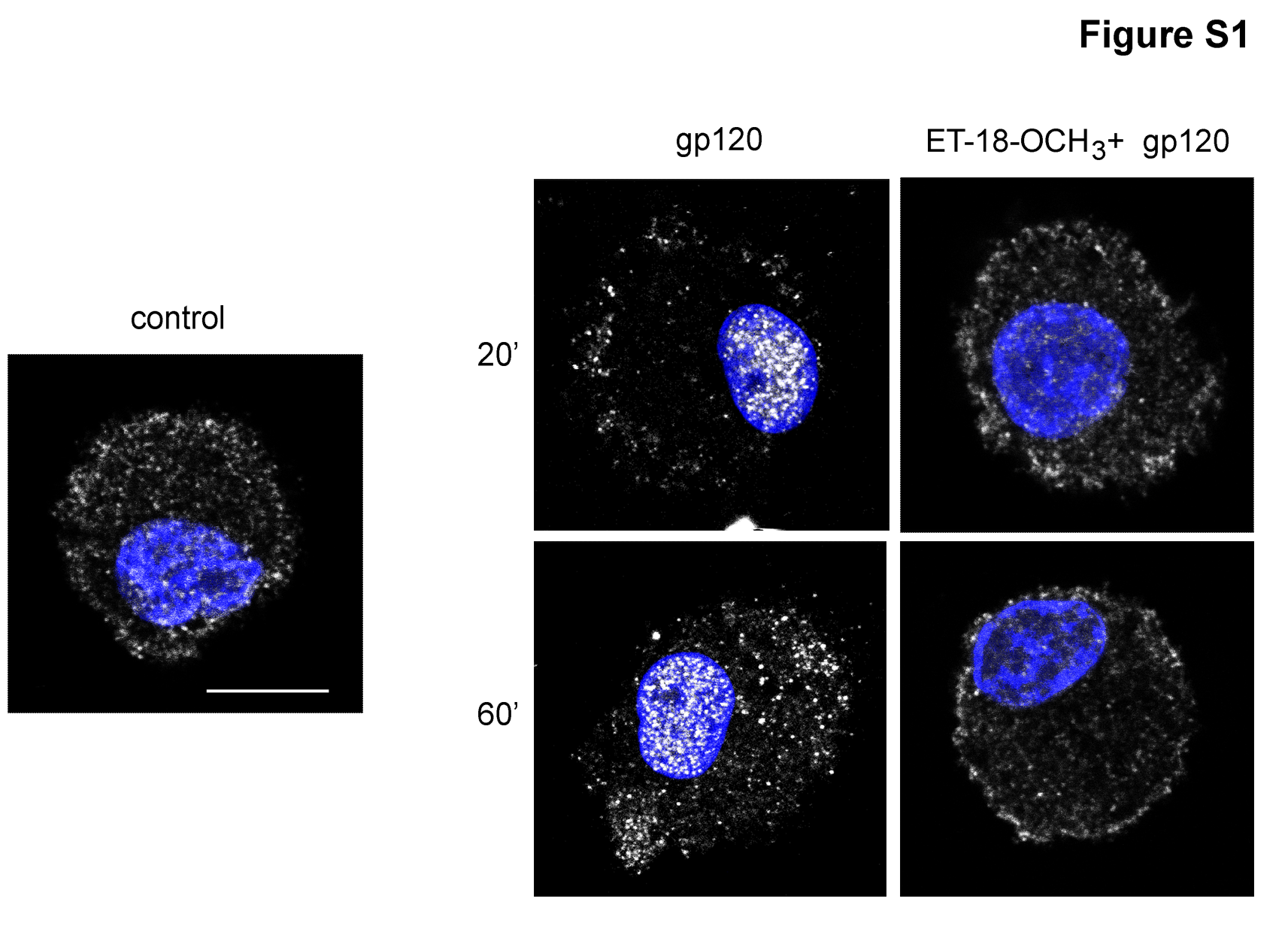

Supplement: Figure S1 — Time-dependent effect of ET-18-OCH3 on gp120-induced PI-PLC β1 nuclear localization in MDM. MDM were treated with ET-18-OCH3 (10 µM) or left untreated (control). After 1 hour, some cultures were exposed to R5 gp120 (3 µg/ml) for the indicated time periods. Cells were then fixed, permeabilized, stained with anti-PI-PLC β1 Ab (pseudocolor gray) and examined by CLSM. Nuclei are reported in blue (DAPI). Representative examples of 3 independent experiments are shown. The bars correspond to 10 µm. (TIF) [file pone.0059705.s001.tif]

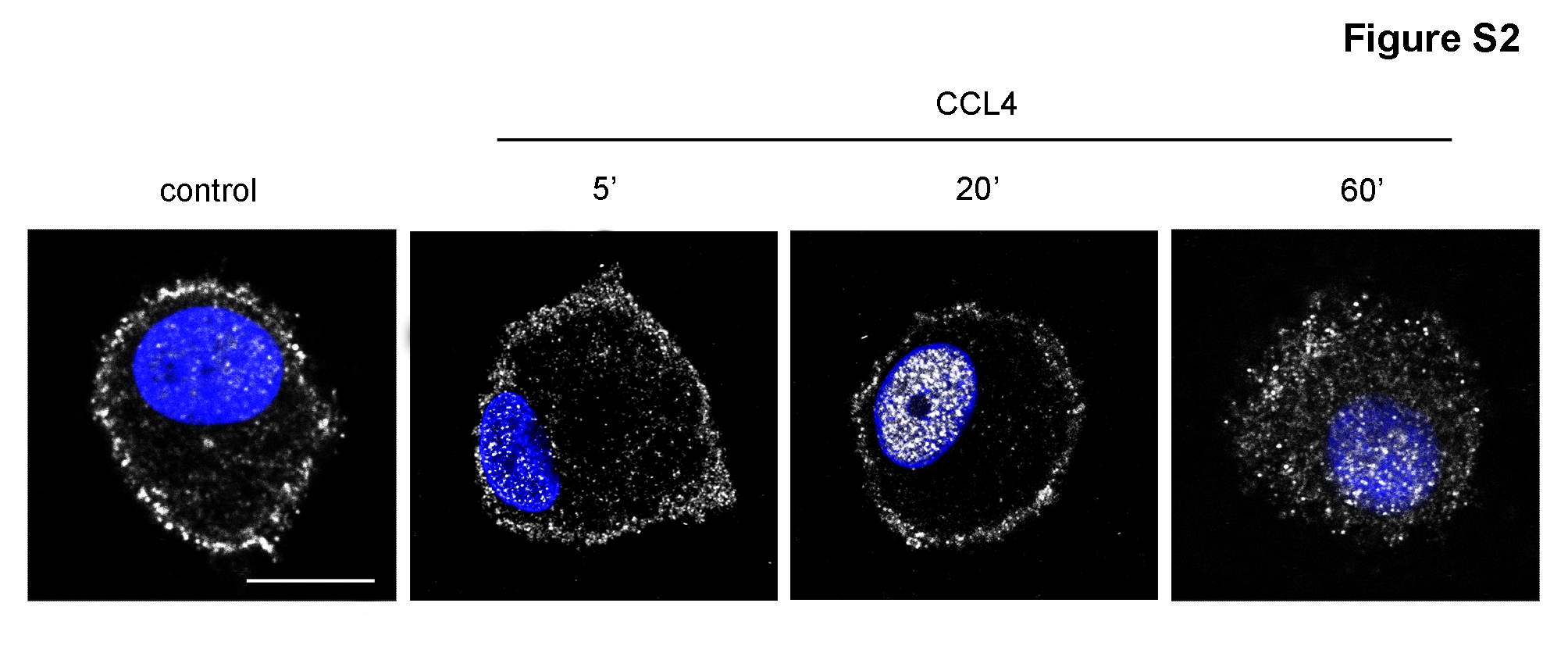

Supplement: Figure S2 — Time-dependent effect of CCL4 on PI-PLC β1 nuclear localization in MDM. MDM were treated with CCL4 (200 ng/ml) for the indicated time periods or left untreated (control). Cells were then fixed, permeabilized, stained with anti-PI-PLC β1 Ab (pseudocolor gray) and examined by CLSM. Nuclei are reported in blue (DAPI). A representative experiment of 4 independently performed is shown. The bars correspond to 10 µm. (TIF) [file pone.0059705.s002.tif]

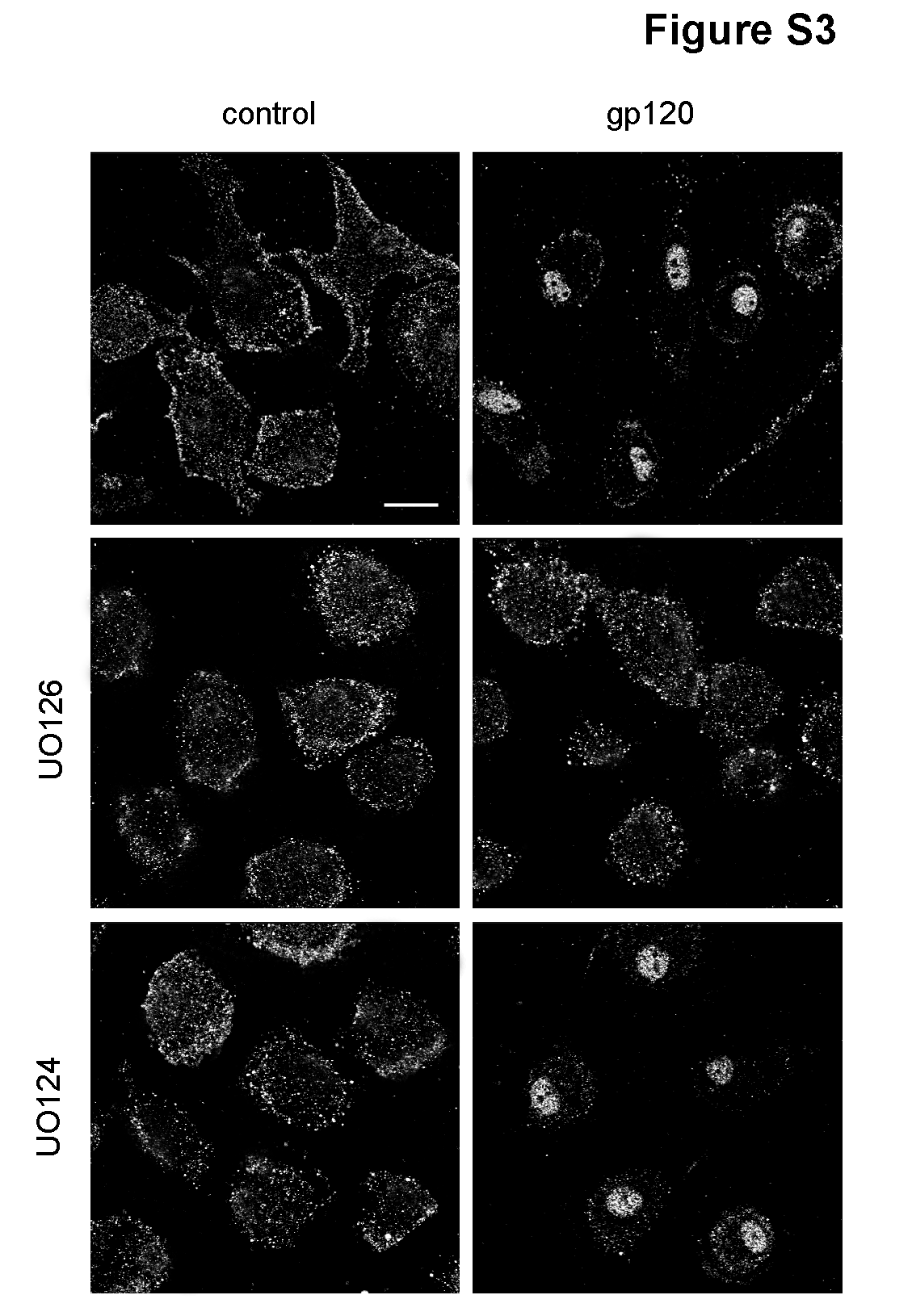

Supplement: Figure S3 — The ERK1/2 inhibitor U0126 abrogates gp120-induced PI-PLC β1 nuclear localization in MDM. MDM were treated with U0126 or its inactive analog U0124 (10 µM) or left untreated (control). After 1 hour, some cultures were exposed to R5 gp120 (3 µg/ml) for 20 minutes. Cells were then fixed, permeabilized, stained with anti-PI-PLC β1 Ab (showed in pseudocolor gray) and examined by CLSM. Panels are representative of 3 independent experiments. The bars correspond to 20 µm. (TIF) [file pone.0059705.s003.tif]

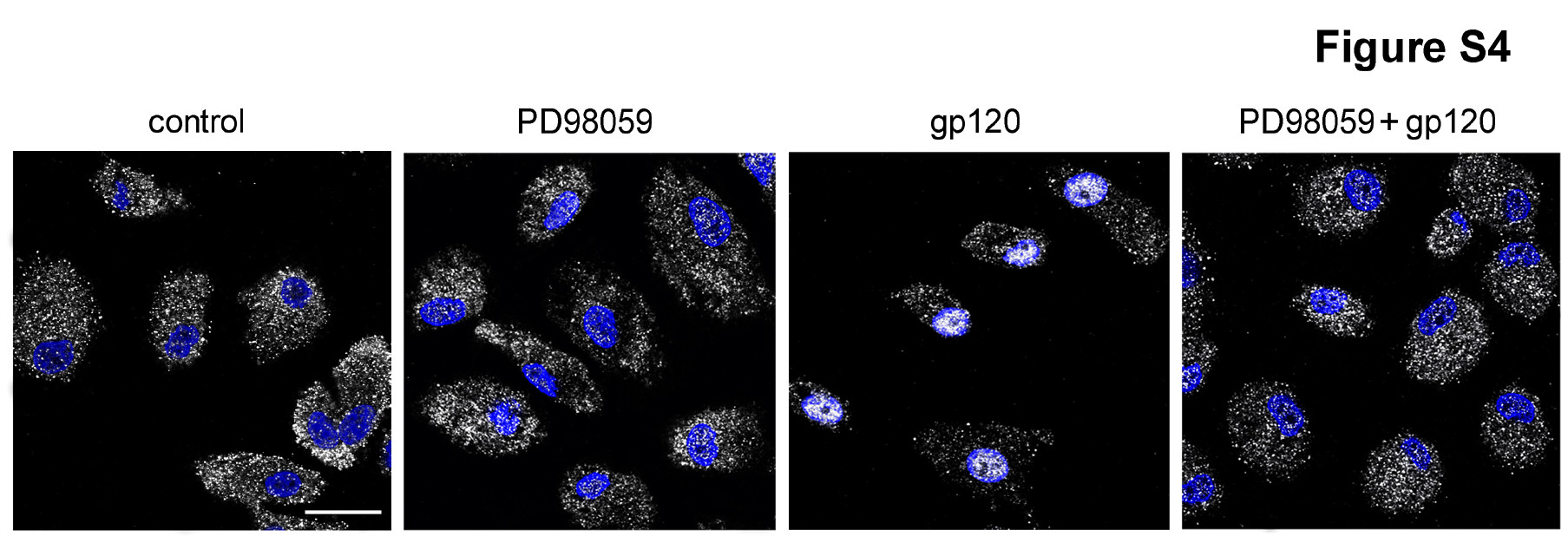

Supplement: Figure S4 — PD98059 inhibits gp120-induced NF-kB p65 subunit nuclear translocation in MDM. MDM were treated with PD98059 (10 µM) or left untreated (control). After 1 hour, some cultures were exposed to R5 gp120 (3 µg/ml) for 1 hour. Cells were then fixed, permeabilized and stained with rabbit anti-p65 Ab (showed in pseudocolor gray) and examined by CLSM analysis. Nuclei are reported in blue (DAPI). The results are representative of 4 independent experiments. The bars correspond to 20 µm. (TIF) [file pone.0059705.s004.tif]

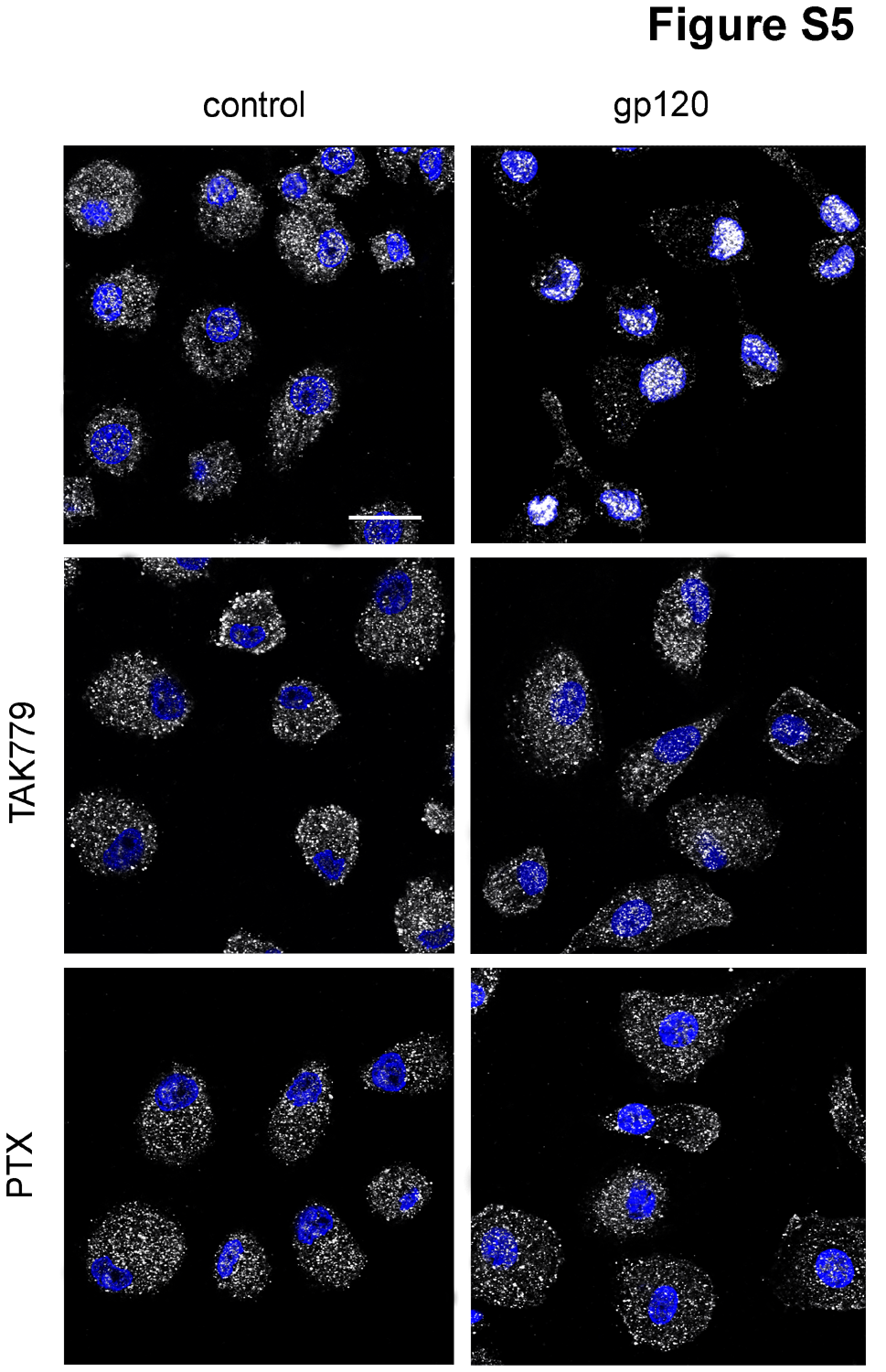

Supplement: Figure S5 — Tak779 and PTX prevent gp120-induced NF-kB p65 subunit nuclear translocation in MDM. MDM were treated with PTX (10 ng/ml) for 2 hours or Tak779 (5 µM) for 1 hour prior to R5 gp120 (3 µg/ml) exposure for 1 hour or left untreated (control). Cells were then fixed, permeabilized and stained with rabbit anti-p65 Ab (showed in pseudocolor gray) and examined by CLSM analysis. Nuclei are reported in blue (DAPI). The results are representative of 2 independent experiments. The bars correspond to 20 µm. (TIF) [file pone.0059705.s005.tif]

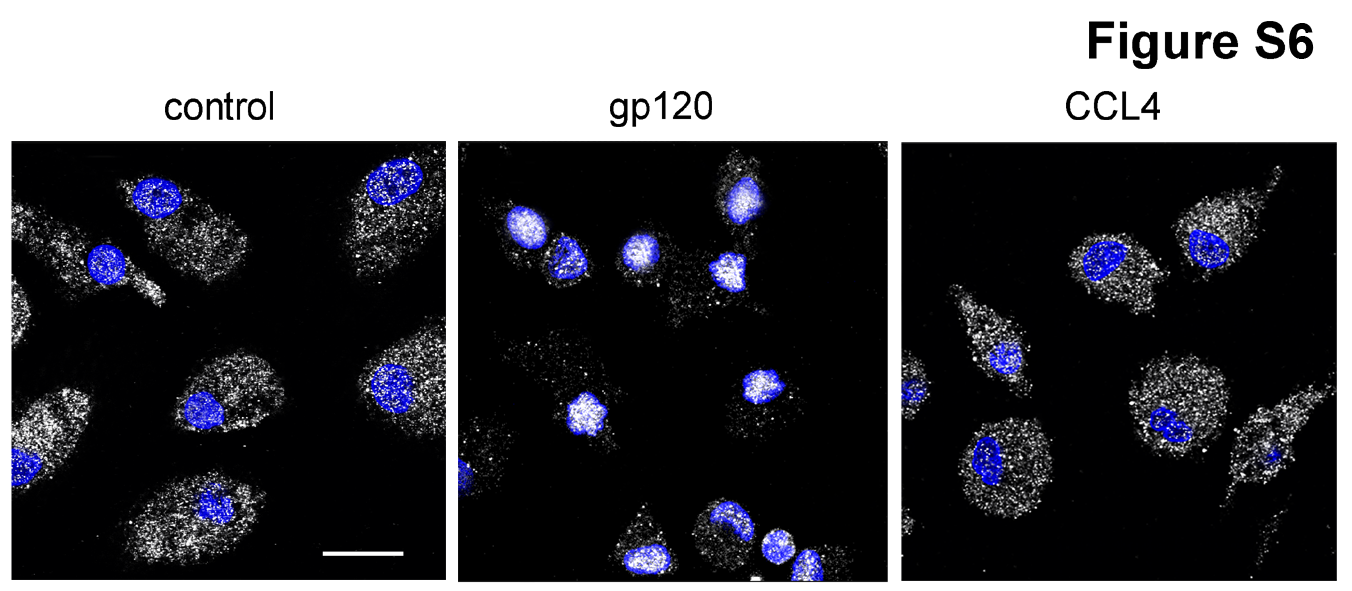

Supplement: Figure S6 — CCL4 does not induce NF-kB p65 subunit nuclear translocation in MDM. MDM were treated with R5 gp120 (3 µg/ml) or CCL4 (200 ng/ml) or left untreated (control). After 1 hour, cells were fixed, permeabilized and stained with rabbit anti-p65 Ab (showed in pseudocolor gray) and examined by CLSM analysis. Nuclei are reported in blue (DAPI). Panels are representative of 2 independent experiments. The bars correspond to 20 µm. (TIF) [file pone.0059705.s006.tif]
